# Hearing loss and incident dementia over 8 years in Black and White older adults: the Atherosclerosis Risk in Communities Neurocognitive Study

**DOI:** 10.3389/fepid.2026.1798451

**Published:** 2026-04-09

**Authors:** Jennifer A. Deal, John J. Shin, Kening Jiang, A. Richey Sharrett, Josef Coresh, Rebecca F. Gottesman, David S. Knopman, Thomas Mosley, Keenan A. Walker, Frank R. Lin, Nicholas S. Reed

**Affiliations:** 1 Department of Epidemiology, Johns Hopkins Bloomberg School of Public Health, Baltimore, MD, United States; 2 Cochlear Center for Hearing and Public Health, Johns Hopkins Bloomberg School of Public Health, Baltimore, MD, United States; 3 Department of Otolaryngology-Head and Neck Surgery, Johns Hopkins School of Medicine, Baltimore, MD, United States; 4 The Dartmouth Institute for Health Policy and Clinical Practice, Geisel School of Medicine at Dartmouth, Lebanon/Hanover, NH, United States; 5 Optimal Aging Institute, New York University Grossman School of Medicine, New York, NY, United States; 6 Stroke Branch, National Institute of Neurological Disorders and Stroke Intramural Research Program, National Institute of Health, Bethesda, MD, United States; 7 Department of Neurology, Mayo Clinic, Rochester, MN, United States; 8 The MIND Center, University of Mississippi Medical Center, Jackson, MS, United States; 9 Laboratory of Behavioral Neuroscience, National Institute on Aging, Intramural Research Program, Baltimore, MD, United States

**Keywords:** dementia, equity, prevention, risk factors, sensory health, underrepresented populations

## Abstract

**Objective:**

We investigated potential racial disparities in the effects of audiometric hearing loss and its treatment on dementia and mortality among 3,602 older adults aged 68–89 years, 22% of whom were self-identified Black race.

**Methods:**

Adjudicated all-cause dementia was determined using neurocognitive test data, proxy reports, and surveillance of hospital records and death certificates. Audiometric hearing loss, defined as the better-ear averaged pure-tone threshold (0.5–4 kHz), was categorized using clinical cutpoints. Multivariable-adjusted Cox proportional hazards models included hearing loss–race interaction terms.

**Results:**

Dementia risk associated with moderate-to-severe hearing loss did not differ by race [Black participants: hazard ratio (HR): 1.66; 95% confidence interval (CI): 1.05, 2.61; White participants: HR: 1.71; 95% CI: 1.16, 2.51; *P*-interaction = 0.92]. However, moderate-to-severe hearing loss was associated with a 2.3-fold increase in mortality among Black participants only (95% CI: 1.17, 4.60).

**Conclusions:**

Our findings emphasize the importance of including minoritized populations in hearing treatment research to build an evidence base for policy development and clinical decision-making. Hearing loss affects the health of both Black and White Americans. Racial disparities in hearing healthcare should be addressed to advance health equity for all older adults.

## Introduction

Black older adults are at greater risk for Alzheimer's disease and related dementias (ADRD) (1) but are less likely to be diagnosed than White adults (2). Understanding how ADRD health determinants differ by race/ethnicity is essential for addressing disparities and advancing health equity among minoritized racial populations. Hearing loss is an established risk factor for ADRD and cognitive decline, with 7%–9% of global dementia cases potentially preventable through hearing loss treatment (1, 3). However, these estimates are derived from studies involving predominantly White populations (1, 3). Few studies have investigated the intersectionality of hearing loss and racial identity in relation to dementia risk (4–6).

The prevalence of hearing loss is lower among Black individuals compared with White individuals (7), perhaps because skin pigmentation is associated with increased melanin in the inner ear, which may act as free radical scavengers and calcium chelators, thereby protecting against oxidative stress (8). Although biological factors may explain observed racial differences in the prevalence of hearing loss, we do not hypothesize that race itself or associated biological differences would impact the association between hearing loss and dementia. In this context, we view race as a proxy for social and cultural experiences (9, 10)—including those shaped by structural racism—which could potentially affect the magnitude of previously reported associations.

Understanding the combined impact of race and hearing loss on cognitive health is needed, given racial disparities in hearing healthcare (11). The significance of this inequity is underscored by recent randomized clinical trial evidence demonstrating that hearing intervention (hearing aids and aural rehabilitation), compared with a health education control, delayed 3-year cognitive decline by 48% in a subset of the study population (12). On average, the group that benefited was older, had lower baseline cognitive function, lower educational attainment and income, and a higher prevalence of comorbidities. They also experienced faster rates of cognitive decline during follow-up. Although the study reported no differences in the intervention effect by race, it was not powered to detect racial differences and was likely impacted by limited representation, as only 11% of the total population self-identified as Black. However, most Black participants were in the group that benefited from the intervention (12).

Given mixed trial results, well-conducted observational studies with prospective follow-up are needed to document racial disparities in ADRD risk and to evaluate potential differential impacts of hearing treatment. We investigated whether the association between audiometric hearing loss and dementia incidence over a median follow-up of 8 years differs by race in a biracial cohort, the Atherosclerosis Risk in Communities Neurocognitive Study (ARIC-NCS). As hearing loss may also be associated with increased mortality (13, 14), which could prevent observation of dementia diagnoses, we also conducted a competing-risks analysis of hearing loss and mortality. Given the higher prevalence of dementia among Black older adults, we hypothesized that the association between hearing loss and dementia would be stronger and that the estimated effects of treatment would be greater among self-identified Black compared to White participants.

## Materials and methods

### Study population

The ARIC study is an ongoing prospective epidemiologic study of 15,792 individuals aged 45–65 years at baseline (1987–1989) from four U.S. communities: Minneapolis, Minnesota; Jackson, Mississippi; Washington County, Maryland; and Forsyth County, North Carolina. This analysis uses data from the ARIC-NCS, which has been ongoing since 2011–2013 (15). We obtained data from up to four clinic visits (visit 5: 2011–2013; visit 6: 2016–2017; visit 7: 2018–2019; and visit 8: 2019–2020). Home/long-term care visits were offered to participants who were unable to come to the clinic. Participants were also contacted annually/semiannually via telephone between clinic visits. Written informed consent was obtained from either the participant or a designated proxy (legally authorized representative, spouse, adult child, adult sibling, friend, or other relative), with written participant assent if the participant was unable to provide consent (e.g., dementia diagnosis). Study procedures were approved by the Institutional Review Board at each site (15).

Of the 15,792 ARIC participants, 9,008 (57%) were alive and free of a dementia diagnosis at visit 5, which served as the baseline for this analysis. Among these participants, 3,917 (43%) also attended visit 6, when hearing was assessed. A total of 265 participants were excluded due to missingness of all audiometric thresholds. Additional exclusions were made for participants missing all cognitive measures (*n* = 22) or education data (*n* = 6); furthermore, following recommendations from ARIC investigators due to concerns about limited representation (16), participants were excluded if their race was other than Black or White (*n* = 7 Asian; *n* = 2 American Indian or Alaska Native) or if they were African American from Minneapolis (*n* = 3) or Maryland (*n* = 8). Two individuals were excluded because their pure-tone average exceeded 90 decibels hearing level (dB HL) in the better ear (7). The small number of such cases limited the ability to infer about this group, and one of the two individuals was an outlier (better-ear pure tone average = 102.5) that unduly influenced regression results. Our analytic sample included 3,602 participants. Compared with participants included in the study, those who were excluded from the analysis were, on average, older and more likely to have less than a high school education, current smoking, hypertension, and diabetes. Among the excluded participants, Black participants were more likely than White participants to have at least one apolipoprotein e4 allele, as well as diabetes and hypertension (Supplementary Table 1).

### Dementia diagnosis

For participants who survived and attended visit 8 (2019–2020), the last clinic visit included in this analysis, standardized algorithmic dementia diagnoses (17) were based on longitudinal cognitive data, the Clinical Dementia Rating Scale, and the Functional Activities Questionnaire. These algorithmic diagnoses were confirmed by expert panel review. For participants who did not attend clinic visits, diagnoses were based on ancillary cognitive information [Telephone Interview for Cognitive Status (visit 5 only), the six-item screener, and the Alzheimer's disease (AD) 8 Dementia Screening Interview (since 2015)] or, if these data were not available, on hospital/death certificate codes. Active surveillance using hospital/death certificate dementia codes was conducted from the date of last participant contact until the date of event or administrative censoring (31 December 2020) and was confirmed via proxy interviews when possible. Given these multiple data sources, to minimize the potential impact of selective attrition in the cohort, dementia diagnoses were completed for all participants, including those who died or were lost to follow-up. When multiple sources of information were available for a given participant, the source with the highest diagnostic confidence was prioritized in the following order: (1) reviewer diagnosis based on an in-person cognitive evaluation, (2) algorithmic diagnosis based on an in-person cognitive evaluation, (3) reviewer diagnosis based on a telephone-based cognitive evaluation, (4) algorithmic diagnosis based on a telephone-based cognitive evaluation, (5) education-adjusted TICS, (6) CDR and FAQ from an informant interview, (7) AD8 result, (8) two SIS results, (9) one SIS result if the participant was lost to follow-up or deceased, (10) hospitalization discharge codes, and (11) death certificate codes. Once dementia was diagnosed, the date of incident dementia was determined according to the following order of priority: (1) the date of the in-person or phone-based cognitive evaluation or the date of the hospitalization discharge record if the latter is earlier, (2) the date of the earliest informant interview, education-adjusted TICS, AD8, or SIS that detected dementia, and (3) the date of death (16). The data sources for dementia diagnoses in the current study do not vary by race (Supplementary Table 2). For diagnoses ascertained by codes, given the expected lag in ascertainment, the date of dementia onset was estimated to be 6 months before the hospitalization or death (18).

### Hearing loss

Hearing loss was measured using pure-tone air conduction audiometry at visit 6 (2016–2017) using the Interacoustics Equinox 2.0 Diagnostic Audiometer in a sound-treated booth during clinic visits or with the ShoeBox portable audiometer during home or long-term care facility visits. A pure-tone average was calculated for thresholds at 0.5, 1, 2, and 4 kHz (in dB HL) in both ears, and the better-hearing ear was used for analysis. Hearing loss was categorized using clinical cutpoints commonly applied in population-based research: ≤25 dB HL (no hearing loss), 26–40 dB HL (mild loss), 41–90 dB HL (moderate-severe loss) (7), with a sensitivity analysis to investigate the current WHO definition, which was updated in 2021 (19).

### Other variables

Race was self-reported at visit 1 (1987–1989) as White, Black, American Indian or Alaska Native, or Asian or Pacific Islander. Because only a small number of participants who were alive and dementia-free at visit 5 (baseline for this analysis) reported American Indian or Alaska Native (*n* = 2) or Asian or Pacific Islander (*n* = 7), we restricted the analysis to White and Black participants. Age (years), sex (male/female), and education (less than high school, high school or equivalent, or greater than high school) were self-reported.

Apolipoprotein E (*APOE*) *ε*4 genotyping was conducted using the TaqMan assay (Applied Biosystems, Foster City, CA, USA) and categorized as 0 vs. ≥1 *ε*4 allele. Smoking status was self-reported as never, former, or current. Body mass index (BMI, kg/m^2^) was calculated from measured height and weight. Diabetes was considered present if at least one of the following conditions was met: fasting blood glucose level ≥126 mg/dL, non-fasting ≥200 mg/dL, use of diabetes medication, or self-reported physician diagnosis. Hypertension was considered present if at least one of the following conditions was met: diastolic blood pressure ≥90 mmHg, systolic blood pressure ≥140 mmHg, or use of hypertensive medications. Prevalent stroke was defined as a self-reported history of physician-diagnosed stroke at visit 1, which was adjudicated through our analytic baseline. Hearing aid use and occupational noise exposure (exposure to very loud noise at a job for 10 or more hours per week) were self-reported. Race, sex, and education were measured at visit 1. All other covariates were defined at visit 5, except for hearing aid use and occupational noise exposure, which were collected at visit 6.

### Statistical analysis

To describe the study groups, the distributions of participant characteristics were summarized by hearing loss status and race. For inferential analysis, multivariable-adjusted Cox proportional hazards models with the Efron method (20) to handle ties were used to estimate hazard ratios and 95% confidence intervals for incident dementia (dependent variable) according to hearing loss status (independent variable). The proportional hazards assumption was verified using Schoenfeld residuals (21). All models were adjusted for covariates, including age, sex, race, education, *APOE ε*4 status, smoking, BMI, occupational noise exposure, diabetes, hypertension, stroke, and hearing aid use. Because individuals with normal hearing are unlikely to use hearing aids, adjustment for hearing aid use was through an interaction term between hearing loss and hearing aid use, without a main effect for hearing aid use. To test whether the association between hearing loss and dementia differed by race, we included an interaction term between hearing loss and race (Black/White) in the model, in addition to the main effect for race. Missing data were imputed using multiple imputation with chained equations, generating 20 sets of imputations (see the Supplementary Material).

A competing-risks analysis of hearing loss and death without dementia was conducted using multivariable-adjusted Cox proportional hazards models, in which dementia diagnosis before death was treated as a censoring event (22).

To investigate the impact of hearing aid use on ADRD and cognitive decline, we restricted to individuals with hearing loss (23) and included hearing aid use as the exposure of interest in the models described above.

## Results

Overall, the mean age of participants was 74.5 years [standard deviation (SD) 4.7 years]; 59% were women, 22% were Black race, and 65% had hearing loss (Table 1). Among Black participants, 51% had hearing loss, which was predominantly mild (72%). In contrast, 71% of White participants had hearing loss, of which 57% was mild. Only 8% of Black participants with hearing loss reported using hearing aids, compared with 33% of White participants.

**Table 1:** Baseline (2011–13) characteristics of participants by race and hearing loss, the Atherosclerosis Risk in Communities Neurocognitive Study (ARIC-NCS), *N* = 3,602.

Characteristic	Total	Black participants		White participants	
		No hearing loss	Hearing loss	No hearing loss	Hearing loss
	*N* = 3,602	*N* = 396	*N* = 408	*N* = 784	*N* = 1,940
Age (years), mean (SD)	74.5 (4.7)	72.6 (3.7)	75.1 (5.0)	72.8 (3.8)	75.4 (4.8)
Female, *N* (%)	2,141 (59.4)	294 (74.2)	273 (66.9)	569 (72.6)	981 (50.6)
Education, *N* (%)					
Less than high school	413 (11.5)	68 (17.2)	112 (27.5)	44 (5.6)	181 (9.3)
High school or equivalent	1,491 (41.4)	124 (31.1)	114 (27.9)	332 (42.4)	890 (45.9)
Greater than high school	1,698 (47.1)	204 (51.5)	182 (44.6)	408 (52.0)	869 (44.8)
≥1 *APOE ε*4, *N* (%)	966 (28.4)	138 (37.0)	146 (38.1)	195 (26.3)	467 (25.5)
Body mass index (kg/m^2^), mean (SD)	29.0 (5.6)	31.2 (5.9)	31.1 (7.1)	28.0 (5.0)	28.5 (5.2)
Ever smoker, *N* (%)	2,106 (59.4)	208 (53.9)	226 (58.1)	447 (57.5)	1,181 (61.5)
Hypertension, *N* (%)	2,565 (73.2)	338 (87.6)	357 (89.9)	497 (65.4)	1,321 (70.0)
Diabetes, *N* (%)	1,015 (29.8)	157 (43.1)	152 (41.3)	172 (22.7)	512 (27.6)
Stroke, *N* (%)	103 (3.0)	15 (4.1)	19 (5.0)	13 (1.7)	52 (2.8)
Occupational noise exposure, *N* (%)	872 (24.7)	89 (22.5)	108 (26.9)	106 (13.7)	541 (28.6)
Better-ear PTA (dB HL), median (25th percentile—75th percentile)	31.3 (22.5, 41.3)	18.8 (15.0, 22.5)	35.0 (29.4, 41.9)	20.0 (16.3, 22.5)	38.8 (31.3, 47.5)
Hearing loss category, *N* (%)					
Normal hearing	1,180 (33.5)	396 (100.0)	–	784 (100.0)	–
Mild hearing loss	1,406 (39.9)	–	295 (72.3)	–	1,111 (57.3)
Moderate-to-severe hearing loss	942 (26.7)	–	113 (27.7)	–	829 (42.7)
Hearing aid use, *N* (%)	724 (20.5)	0 (0.0)	31 (7.7)	15 (1.9)	636 (33.6)

*APOE*, apolipoprotein E; dB HL, decibels hearing level; kg/m^2^, kilogram/meter^2^; PTA, pure-tone average; SD, standard deviation. Hearing was measured with pure-tone audiometry, and a four-frequency pure-tone average in the better-hearing ear >25 dB HL was defined as hearing loss. For regression models, participants with hearing loss were categorized as having mild hearing loss if better-ear PTA was >25 dB HL and ≤40 dB HL and as having moderate-to-severe hearing loss if the better-ear PTA was >40 dB HL and ≤90 dB HL. Seven Black participants and 67 White participants had missing data on some, but not all, hearing thresholds and so are excluded from Table 1. These participants were included in regression models after imputing hearing status using multiple imputation.

Compared with participants with normal hearing, those with hearing loss were more likely to be older, male, have a history of occupational noise exposure, and have lower educational attainment (Table 1). During a median follow-up of 8.0 years (range 0.4–9.4 years), 501 (14%) participants developed dementia, of which 147 (29%) were Black, and 376 (79%) had hearing loss. An additional 256 (7%) participants died without dementia during follow-up; among these, 61 (24%) were Black, and 184 (74%) had hearing loss.

Overall, mild and moderate-to-severe hearing loss (vs. normal hearing) were associated with a 49% and 68% increased risk of dementia over 8 years, respectively [hazard ratio (HR) for mild vs. normal:1.49; 95% confidence interval (CI):1.16, 1.92; HR for moderate-to-severe vs. normal:1.68; 95% CI:1.24, 2.28] (Table 2). The association was similar for Black and White participants (*P*-interaction = 0.92). In the analysis of death as a competing risk, hearing loss was associated with dementia-free mortality among Black participants (HR for moderate-to-severe hearing loss vs. normal hearing: 2.32; 95% CI: 1.17, 4.60) but not among White participants (HR: 1.12; 95% CI: 0.70, 1.78; *P*-interaction = 0.07) (Table 3). Results were robust to the use of the most recent WHO cutpoints (18) to define hearing loss (Supplementary Table 3).

**Table 2:** Multivariable-adjusted hazard ratios (HRs) and 95% confidence intervals (CIs) of incident dementia (2011−2013 to 2019–2020) by hearing loss (2016–2017) and race, the Atherosclerosis Risk in Communities Neurocognitive Study (ARIC-NCS), *N* = 3,602.

Population	No hearing loss		Hearing loss category				*P*-trend
			Mild		Moderate-to-severe		
	*N*_Dementia_/*N*_Total_	HR (95% CI)	*N*_Dementia_/*N*_Total_	HR (95% CI)	*N*_Dementia_/*N*_Total_	HR (95% CI)	
Total	103/1,180	Referent	203/1,406	**1.49 (1.16, 1.92)**	173/942	**1.68 (1.24, 2.28)**	**0**.**009**
Black	51/396	Referent	63/295	1.40 (0.96, 2.03)	29/113	**1.66 (1.05, 2.61)**	**0**.**018**
White	52/784	Referent	140/1,111	**1.57 (1.13, 2.18)**	144/829	**1.71 (1.16, 2.51)**	**0**.**006**
*P*-interaction				0.648		0.916	

Estimates are from a Cox proportional hazards model adjusted for age, sex, education, race, *APOE* ε4, smoking, body mass index, noise exposure, diabetes, hypertension, stroke, and hearing aid use. An interaction term between race and hearing loss categories was used to estimate associations in each race group. Hearing was measured using pure-tone audiometry. Participants were categorized as having mild hearing loss if the better-ear PTA was >25 decibels hearing level (dB HL) and ≤40 dB HL and as having moderate-to-severe hearing loss if the better-ear PTA was>40 dB HL and ≤90 dB HL. The total number of individuals reported in the table does not equal 3,602 (the total sample) because seven Black participants and 67 White participants had missing data on some, but not all, hearing thresholds; these participants are included in the regression models after imputing hearing status using multiple imputation. The *P*-trend value was obtained by modeling hearing loss categories as a continuous variable and is consistent with stronger associations between hearing loss and dementia as the severity of hearing loss increases. The *P*-interaction value is from the interaction term between race and hearing loss included in the model. Estimates in boldface are statistically significant at the alpha = 0.05 level.

**Table 3:** Multivariable-adjusted hazard ratios (HRs) and 95% confidence intervals (CIs) of death without dementia (2011–13 to 2019–20) by hearing loss (2016–17) and race, the Atherosclerosis Risk in Communities (ARIC) Study, *N* = 3,602.

Population	No hearing loss		Hearing loss category				*P*-trend
			Mild		Moderate-Severe		
	*N*_Death_/*N*_Total_	HR (95% CI)	*N*_Death_/*N*_Total_	HR (95% CI)	*N*_Death_/*N*_Total_	HR (95% CI)	
Total	64/1,180	Referent	93/1,313	1.02 (0.73, 1.43)	91/942	1.40 (0.93, 2.11)	**0**.**009**
Black	20/396	Referent	25/295	1.48 (0.81, 2.70)	15/113	**2.32 (1.17, 4.60)**	**0**.**018**
White	44/784	Referent	68/1,111	0.84 (0.57, 1.25)	76/829	1.12 (0.70, 1.78)	**0**.**006**
*P*-interaction				0.117		0.071	

Estimates for this competing-risks analysis are from a Cox proportional hazards model, with dementia before death treated as a censoring event. The model was adjusted for age, sex, education, race, *APOE* ε4, smoking, body mass index, noise exposure, diabetes, hypertension, stroke, and hearing aid use. An interaction term between race and hearing loss categories was used to estimate associations in each race group. Hearing was measured using pure-tone audiometry. Participants were categorized as having mild hearing loss if the better-ear PTA was >25 decibels hearing level (dB HL) and ≤40 dB HL and as having moderate-to-severe hearing loss if the better-ear PTA was >40 dB HL and ≤90 dB HL. The total number of individuals reported in the table does not equal 3,602 (the total sample) because seven Black participants and 67 White participants had missing data on some, but not all, hearing thresholds; these participants are included in the regression models after hearing status was imputed using multiple imputation. The *P*-trend value was obtained by modeling hearing loss categories as a continuous variable and is consistent with stronger associations between hearing loss and mortality as the severity of hearing loss increases. The *P*-interaction value is from the interaction term between race and hearing loss included in the model. Estimates in boldface are statistically significant at the alpha = 0.05 level.

When the analysis was restricted to the 2,348 participants with hearing loss, hearing aid use vs. non-use did not statistically significantly impact dementia risk over time, and no differences were observed by race (Table 4).

**Table 4:** Multivariable-adjusted hazard ratios (HRs) and 95% confidence intervals (CIs) of dementia (2011–2013 to 2019–2020) by hearing aid use (2016–2017) and race among individuals with hearing loss, the Atherosclerosis Risk in Communities (ARIC) Study, *N* = 2,348.

Population	No hearing aid use		Hearing aid use	
	*N*_Dementia_/*N*_Total_	HR (95% CI)	*N*_Dementia_/*N*_Total_	HR (95% CI)
Total	257/1,629	Referent	97/667	0.87 (0.68, 1.12)
Black	86/370	Referent	5/31	0.78 (0.31, 1.96)
White	171/1,259	Referent	92/636	0.88 (0.68, 1.13)
*P*-interaction				0.800

Estimates are from a Cox proportional hazards model adjusted for age, sex, education, race, *APOE* ε4, smoking, body mass index, noise exposure, diabetes, hypertension, and stroke. An interaction term between race and hearing aid use was used to estimate associations in each race group. Hearing was measured using pure-tone audiometry. This analysis was restricted to people with hearing loss (better-ear pure-tone average >25 decibels hearing level), as individuals without hearing loss are not at risk for cognitive decline due to hearing loss and therefore would not be expected to benefit from hearing aid use. The total number of individuals reported in the table does not equal 2,348 (the total sample) because seven Black participants and 45 White participants had missing data on hearing aid use; these participants are included in the regression models after hearing aid use was imputed using multiple imputation. Estimates below zero suggest a protective effect of hearing aid use among individuals with hearing loss. The *P*-interaction value is from the interaction term between race and hearing aid use included in the model.

No associations are statistically significant at the alpha = 0.05 level.

## Conclusions

In this study of 3,602 men and women aged 66–90 years (22% Black) from four U.S. communities, hearing loss was independently associated with a 55% increased risk of all-cause dementia. The risk increased with greater severity of hearing loss. We did not find differences in estimated associations by racial identity. Importantly, moderate-to-severe hearing loss was associated with a greater than 2-fold increase in the risk of mortality among Black participants. This work contributes to the limited body of research characterizing differences in the association between hearing loss and dementia by race (4), and to our knowledge, this is the first study to report a strong racial disparity in the association between hearing loss and mortality.

Although some prior studies have presented race-stratified results (4) or been restricted to one race/ethnicity group (5), few have examined differences in the association between hearing loss and dementia by race. In a study of 1,881 participants from the Washington Heights–Inwood Columbia Aging Project [mean age 75.8 (SD 6.3) years, 70% women], hearing difficulty identified by the study examiner was associated with a 69% increased risk of incident dementia over a mean follow-up of 7.4 years. Similar to our study, there was no difference in dementia risk by race/ethnicity (Hispanic, Black, White, *P*-interaction = 0.19) (6).

Our findings are generally consistent with seven studies (24–30) identified in prior methodologically rigorous systematic reviews (1, 3) and two other studies that applied the same robust inclusion/exclusion criteria (“following a cohort of cognitively healthy people for at least 5 years; using an objective measure of peripheral hearing (pure-tone audiometry); looking at incident dementia as an outcome; and adjusting for age and cardiovascular risk factors as potential confounding factors”) (3, 31, 32). All but one study (24) reported positive associations between hearing loss and increased dementia risk.

Five of the nine total studies did not include Black participants (24–26, 31, 32). Among the studies that did include Black participants (27–30), three were conducted within the same cohort (28–30). In a study of 639 participants aged 36–90 years from the Baltimore Longitudinal Study on Aging with 8% Black participants and adjusting for race, there were 1.89-fold and 3.00-fold increases in the hazard of all-cause dementia over 12 years for mild and moderate (vs. normal hearing) hearing loss, respectively (27). Three studies were conducted in the U.S. Health Aging and Body Composition (Health ABC) Study, the only study with a proportion of Black participants similar to our study (33%). In one study, moderate-to-severe vs. normal hearing was associated with a 55% increase in the 9-year risk hazard of incident dementia among 1,889 participants (mean age 75 years); however, unlike our study, mild hearing loss was not statistically significantly associated with increased risk (28). Two additional studies using data from the same cohort (Health ABC) focused on multiple sensory impairment (29) and the combined effects of hearing loss and depressive symptoms on dementia risk (30), but both reported findings for hearing loss alone, with estimates indicating 25% (29) to 42% (30) increased dementia risk associated with hearing loss. Differences in analytic samples (due to the different goals of the analyses) and adjustment factors may explain the variation in estimates within the same cohort; the lowest estimate (25%) additionally adjusted for cardiovascular disease (myocardial infarction, congestive heart failure, etc.), transient ischemic attack, alcohol use, and physical activity (29). Our study includes a larger sample size and a greater absolute number of Black participants than the Health ABC study [*N* = 811 vs. *N* = 625 (28)] but is similar in terms of age distribution and follow-up duration. Our estimated effect size for the total population, after adjusting for race, was most consistent with the original report, which found a 55% increased risk of dementia associated with hearing loss (28) (compared with a 49%–68% increased risk in our study). In summary, although the association between hearing loss and dementia has been investigated in many studies, the number of studies using rigorous methods—including objectively measured hearing loss—remains small, and the racial diversity within those studies is even more limited.

To investigate the etiologic association between hearing loss and dementia, our primary analysis estimated the cause-specific hazard of dementia before death according to hearing loss status. Contrary to our study hypothesis, we did not observe differences in estimates by race. This finding may indicate that there is no relative difference in dementia risk associated with hearing loss by race. Alternatively, we may have been underpowered to detect a difference; however, race-stratified estimates are qualitatively very similar. Importantly, our results cannot be interpreted as the predicted risk of dementia over time because the occurrence of dementia depends not only on dementia risk but also on competing events such as death. To aid in the interpretation of our findings, we therefore conducted a secondary analysis to estimate the cause-specific hazard of death without dementia. Because the associations were stronger than the estimated associations for the dementia analysis among Black participants only, it is possible that differential survival by hearing loss status had a substantial impact on the duration of time Black participants remained at risk for dementia compared with White participants.

Hearing loss is thought to increase the risk of dementia and cognitive decline through several causal mechanisms (33). Degraded auditory signals may require greater processing effort, leading to increased cognitive load, which can negatively affect performance on cognitive tasks, particularly those related to working memory and executive function. Individuals with hearing loss may recruit executive networks (34) and exhibit cross-modal plasticity between the somatosensory and auditory systems (35) for compensatory processing of degraded acoustic signals.  Hearing loss is also associated with lower gray matter volume in the primary auditory cortex (34), faster rates of brain atrophy over time in the temporal lobe and whole brain (36, 37), and poorer white matter microstructural integrity in the temporal lobe and limbic tracts (38). Hearing loss may also increase social isolation and loneliness (39), both of which are risk factors for dementia (1).

It is also possible that documented associations in the observational literature do not reflect a causal relationship but instead reflect a common underlying pathology (e.g., systemic vascular disease affecting both the brain and the ear). Our results remained robust after adjustment for demographic and clinical covariates, including cardiovascular factors, although we cannot rule out the possibility of associations due to unmeasured confounding (e.g., inflammation, genetic factors). Associations could be due to other sources of bias. For example, if individuals with hearing loss are unable to hear instructions during cognitive testing used for dementia adjudication, their performance might reflect their hearing ability rather than their cognitive function. However, to ensure that all participants can register auditory test items, we confirm speech audibility prior to neurocognitive assessment using a standardized protocol (40). In addition, only three of 10 tests in the cognitive battery (Logical Memory I and II, Digit Span Backwards) are administered aurally, and prior work in this cohort has shown that the associations with hearing loss are not due solely to performance on these tests (41).

The possible pathways thought to link hearing loss to mortality include etiologic contributors(such as cognitive decline and dementia, social isolation and loneliness, falls, frailty, mood disorders, accidents (motor vehicle, work-related), and potentially increased cardiovascular disease) or common cause (e.g., residual confounding from clinical cardiovascular disease) (42). A systematic review and meta-analysis reported that hearing loss was associated with a 13% increased risk of all-cause and 28% increased risk of cardiovascular mortality; however, there were too few studies to investigate whether hearing aid use could mitigate these associations (42).

If the pathways linking hearing loss to cognitive health and mortality are causal, hearing loss treatment might be beneficial for these outcomes. In our study, self-reported hearing aid use was not associated with delayed dementia diagnosis. The large-scale Aging and Cognitive Health Evaluation in Elders (ACHIEVE) randomized controlled trial evaluated the effect of best-practice hearing rehabilitation compared with a health education control on 3-year cognitive decline, using the same neurocognitive test battery as our study (12). The ACHIEVE trial was nested within the ARIC-NCS, with some participants recruited from ARIC-NCS and other recruited *de novo* from the community. Overall, the findings were null; however, a prespecified sensitivity analysis found that hearing treatment reduced 3-year cognitive decline by 48% among the 238 participants recruited from the ARIC-NCS trial. Continued follow-up of the ACHIEVE cohort will yield insights into the long-term cognitive effects of hearing aid use. However, it should be noted that the hearing intervention in ACHIEVE involved more than simply providing hearing aids (12, 43); therefore, direct comparisons between the results from that study and our findings may be misleading. Additional research is needed to determine whether hearing aids alone are sufficient to impact cognitive health or whether rehabilitative services, such as those provided in ACHIEVE, are needed to see benefit. However, our estimates trended in the expected direction, suggesting a 13%–22% decrease in the risk of ADRD associated with hearing aid use. Given the smaller sample size of individuals who used hearing aids in our study, particularly Black participants, we were underpowered to detect small-to-medium associations; only 31 Black individuals with hearing loss reported using hearing aids, of whom five received a dementia diagnosis during follow-up. Overall, our findings support the importance of hearing health for the cognitive and overall health of Black and White Americans and highlight the need for additional research to identify who may benefit from treatment. Critically, future studies must ensure adequate representation of minoritized racial populations to ensure sufficient sample size and power for hypothesis testing.

Limitations of our study include the fact that hearing was not assessed until visit 6, 5 years after baseline. However, we believe that our approach is valid, given that hearing change generally progresses slowly over time (1–2 dB per year) and that hearing loss was measured in the better-hearing ear (most medical conditions that acutely affect hearing are rare and do not affect hearing bilaterally). In addition, it is unlikely that subclinical cognitive impairment would affect the reliability of audiometric testing (i.e., reverse causation). PTA is a measure of the auditory periphery and thus relies on cochlear transduction and neuronal afferents to brainstem nuclei, without significant higher auditory cortical processing, and valid hearing thresholds can be obtained in persons with dementia (44). Inclusion of visit 5 data allows for a follow-up duration long enough to observe sufficient dementia events for analysis and addresses the concern that cognitive impairment measured at one time is more susceptible to confounding due to education and other social factors than analyses of change over time. Second, although the availability of multiple sources of data and the ability to assign dementia diagnoses for all participants—including those who died or were lost to follow-up—is a strength of our study, a potential limitation is that auxiliary cognitive information (telephone assessments, hospital and death certificate codes) is likely insensitive for dementia diagnosis compared with in-person evaluations. There may also be a few false-positive hospital codes (17, 18). However, including broader data sources minimized missing data and, thus, reduced potential selection bias due to informative attrition. In addition, the data sources used for diagnosis did not vary by race, minimizing concerns about differential measurement error influencing our study results. A third limitation of our study is that hearing was measured at only one point in time, and we did not have data on the duration of hearing loss. Future studies should incorporate repeated hearing assessments over time to better investigate how changes in hearing are associated with changes in cognition. Furthermore, as with all published studies in this area, the representation of Black individuals was limited. Caution is warranted when generalizing these results, as the inference is based on a small number of individuals. Ongoing studies with large samples of Black older adults and well-measured cognitive outcomes should consider adding hearing measures. Data harmonization efforts across existing studies could also address this concern. Furthermore, although ARIC originally used probability sampling to recruit participants (45), participants who survived to have their hearing measured differed from the original sample in important demographic and clinical characteristics, further limiting the generalizability of our study findings. Finally, because all Black participants were recruited from only two sites—Jackson (92%) and Forsyth County (8%)—we could not disentangle the impact of race from that of geographic location.

Hearing loss is associated with a greater risk of dementia and mortality in older Black and White adults, with a strong disparity in mortality risk among Black adults. Most studies of older adults that include both hearing and cognitive measures are underpowered to examine differences in associations by race, particularly when investigating the impact of hearing aid use. Our findings emphasize the importance of including sufficiently large proportions of self-identified Black participants to investigate possible racial differences in hearing loss (and hearing treatment) associations or novel statistical methods (e.g., harmonization) to pool data from multiple studies when possible. Only 29% of Americans with hearing loss use hearing aids (4), and this prevalence is even lower among Black Americans—estimated at 6%–10% (7, 11, 46). Furthermore, although hearing aid use has generally increased in the United States, a strong disparity in uptake remains, with more White Americans adopting hearing aids over time than Black Americans (47). The reasons for this disparity are unknown, but one possibility is that it reflects financial barriers (e.g., hearing device cost). However, a study of 3,054 individuals from the National Health and Aging Trends Study (mean age 80 years, 49% female, 17% Black race) found that, after adjustment for socioeconomic and clinical factors, disparities in hearing aid use persisted across income strata [below the federal poverty level (FPL), between 100% and 200% of the FPL, and above 200% of the FPL] (48), suggesting that the difference is not solely due to differences by race in traditional measures of socioeconomic status, like income. Another possibility is differences in access to healthcare; however, interestingly, a study using data from the National Health and Nutrition Examination Survey found that Black participants were twice as likely as White participants to have undergone recent hearing testing (11). Further research is needed to understand healthcare disparities by race in hearing and should include diverse measures of socioeconomic status and factors associated with racial segregation and discrimination. Overall, our findings highlight the importance of addressing racial disparities in hearing healthcare to improve the cognitive and overall health of Black older adults in the United States.

## Data Availability

The datasets presented in this study can be found in online repositories. The names of the repository/repositories and accession number(s) can be found below: pre-existing data access policies for the parent cohort study specify that research data requests can be submitted to the steering committee; these will be promptly reviewed for confidentiality or intellectual property restrictions and will not unreasonably be refused. Individual-level patient or protein data may also be restricted by consent, confidentiality, or privacy laws/considerations. These policies apply to both clinical and proteomic data. Additional information on how to obtain data, including access to some data via BioLINCC, which is free of charge and does not require ARIC Study approval, is available at: https://aric.cscc.unc.edu/aric9/researchers/Obtain_Submit_Data.
