# Supplementary material for: Hearing loss and incident dementia over 8 years in Black and White older adults: the Atherosclerosis Risk in Communities Neurocognitive Study
Source: Front Epidemiol. 2026 Apr 9;6:1798451. doi: 10.3389/fepid.2026.1798451 (PMC13102765; doi:10.3389/fepid.2026.1798451)
Supplement: Supplementary file 1 [file Supplementaryfile1.docx]

**Supplemental Material.**

**S1. Missing Data Imputation Methods**

Missing exposure and covariate data were imputed using multiple imputation using chained equations with 20 sets of imputations. The number of missing values imputed for each variable was: hearing loss (n=56), smoking status (n=207), *APOE* ε4 genotype (n=150), body mass index (n=177), history of occupational noise exposure (n=70), diabetes (n=187), hypertension (n=96), history of stroke (n=148) and hearing aid use (n=67). The imputation model included all final covariates included in the analysis as well as auxiliary variables including self-reported hearing in each ear (measured at Visit 6), speech-in-noise performance as measured by the Quick Speech-in-noise test (measured at Visit 6), depressive symptoms as assessed by the Center for Epidemiologic Studies Depression Score (measured at Visits 4, 5 and 6), smoking status (Visit 6), diabetes (Visit 4), fasting glucose (Visit 5), hemoglobin A1c (Visit 5), hypertension (Visit 4), systolic blood pressure (Visit 5), diastolic blood pressure (Visit 6), body mass index (Visits 4 and 6), visit type at Visit 6 (clinic visit or home or long-term care facility visit), global cognitive factor score (Visit 5), self-reported health as compared to peers of the same age (from annual follow-up telephone calls between Visits 4 and 5), and history of coronary heart disease and hospitalization count since Visit 4 (1996-98, collected as part of the annual follow-up telephone calls between Visits 4 and 5).

**S1. Supplemental Table 1.** Visit 1 (1987-89) Characteristics of Participants Included and Excluded in the Analytic Sample by Race, The Atherosclerosis Risk in Communities Neurocognitive Study (ARIC-NCS), N=15,710

|  | **Total** | **Included Participants** | | **Excluded Participants** | |
| --- | --- | --- | --- | --- | --- |
|  |  | **Black Race** | **White Race** | **Black Race** | **White Race** |
|  | **N=15,710** | **N=811** | **N=2,791** | **N=3,448** | **N=8,660** |
| Age (years), Mean (SD) | 54.2 (5.8) | 50.3 (4.6) | 51.0 (4.6) | 54.3 (5.8) | 55.5 (5.6) |
| Female, N (%) | 8,688 (55) | 569 (70) | 1,572 (56) | 2,060 (60) | 4,462 (52) |
| Education, N (%) |  |  |  |  |  |
| Less than High School | 3,764 (24) | 183 (23) | 230 (8) | 1,593 (46) | 1,745 (20) |
| High School or Equivalent | 6399 (41) | 240 (30) | 1,251 (45) | 963 (28) | 3,935 (45) |
| Greater than High School | 5,568 (35) | 388 (48) | 1,310 (47) | 880 (26) | 2,966 (35) |
| ≥ 1 *APOE* ε4, N (%) | 4,619 (31) | 287 (35) | 679 (24) | 1,323 (38) | 2,321 (27) |
| Body Mass Index (kg/m^2^), Mean (SD) | 27.7 (5.4) | 29.2 (5.7) | 26.4 (4.4) | 29.7 (6.3) | 27.2 (5.0) |
| Current Smoker, N (%) | 4,136 (26) | 173 (21) | 423 (15) | 1,102 (32) | 2,420 (28) |
| Hypertension, N (%) | 5,496 (35) | 326 (40) | 432 (15) | 2,044 (59) | 2,685 (31) |
| Diabetes, N (%) | 1,870 (12) | 48 (6) | 95 (3) | 773 (22) | 951 (11) |

Abbreviations: *APOE*, Apolipoprotein E; kg/m^2^, kilogram/meter^2^; SD, Standard Deviation.

**S2. Supplemental Table 2.** Data Sources for Dementia Diagnoses by Race, The Atherosclerosis Risk in Communities Neurocognitive Study (ARIC-NCS), N=501

| **Dementia Data Source** | **Total** | **Black Race** | **White Race** |
| --- | --- | --- | --- |
|  | **N(%)** | **N(%)** | **N(%)** |
| In-person assessment | 311 (62) | 93 (63) | 218 (62) |
| Telephone assessment | 128 (26) | 38 (26) | 90 (25) |
| Hospitalization or death certificate codes | 62 (12) | 16 (11) | 46 (13) |

For participants who survived and attended Visit 8 (2019-20), the last clinic visit included in this analysis, standardized algorithmic dementia diagnoses were based on an in-person assessment including longitudinal cognitive data, the Clinical Dementia Rating Scale, and the Functional Activities Questionnaire. Algorithmic diagnoses were confirmed by expert panel review. For participants who did not attend visits, diagnoses were based on ancillary cognitive information [Telephone Interview for Cognitive Status (Visit 5 only); six-item screener and Alzheimer’s disease (AD) 8 since 2015] or, if not available, on hospital/death certificate codes. Active surveillance through hospital/death certificate dementia codes was conducted from the date of last participant contact through date of event or administrative censoring (December 31, 2020) and confirmed via proxy interviews when possible. When multiple sources of information exist for a given participant, the order of priority utilized to diagnosis incident dementia is (1) reviewer diagnosis based on an in-person cognitive evaluation, (2) algorithmic diagnosis based on an in-person cognitive evaluation, (3) reviewer diagnosis based on a phone-based cognitive evaluation, (4) algorithmic diagnosis based on a phone-based cognitive evaluation, (5) education-adjusted TICS, (6) the CDR and FAQ from an informant interview, (7) AD8 result, (8) two SIS results, (9) one SIS result if the participant is lost to follow up or deceased, (10) hospitalization discharge codes, and (11) death certificate codes. Once dementia has been diagnosed, the order of priority for determining the date of incident dementia is (1) date of in-person or phone-based cognitive evaluation or date of hospitalization discharge record if the latter is earlier, (2) date of the earliest informant interview, education-adjusted TICS, AD8, or SIS that detected dementia, and (3) date of death.

**S3. Supplemental Table 3.** Multivariable-adjusted Hazard Ratios (HR) and 95% Confidence Intervals (CI) of Incident Dementia (2011-13 to 2019-20) by Hearing Loss (WHO definition, 2016-17) and Race, The Atherosclerosis Risk in Communities Neurocognitive Study (ARIC-NCS), N=3,602

| **Population** | **No Hearing Loss** | | **Hearing Loss Category** | | | | ***P*-trend** |
| --- | --- | --- | --- | --- | --- | --- | --- |
|  |  |  | **Mild** | | **Moderate-Severe** | |  |
|  | **N_Dementia_/**  **N_Total_** | **HR (95% CI)** | **N_Dementia_/**  **N_Total_** | **HR (95% CI)** | **N_Dementia_/**  **N_Total_** | **HR (95% CI)** |  |
| **Total** | 41/ 571 | Referent | 183/ 1,493 | **1.42 (1.01, 2.01)** | 277/ 1,538 | **1.78 (1.23, 2.54)** | **0.019** |
| **Black** | 21/ 209 | Referent | 68/ 390 | 1.27 (0.77, 2.08) | 58/ 212 | **1.77 (1.06, 2.96)** | **0.016** |
| **White** | 20/ 362 | Referent | 115/ 1,103 | 1.55 (0.96, 2.52) | 219/ 1,326 | **1.81 (1.11, 2.95)** | **0.029** |
| ***P*-interaction** |  |  |  | 0.563 |  | 0.958 |  |

Estimates are from a Cox proportional hazard model adjusted for age, sex, education, race, *APOE* ε4, smoking, body mass index, noise exposure, diabetes, hypertension, stroke, and hearing aid use. An interaction term between race and hearing loss categories was used to estimate associations in each race group. Hearing was measured with pure tone audiometry. Participants were categorized as having mild hearing loss if better-ear PTA ≥20 decibel hearing level (dB HL) and <35 dB HL and having moderate or greater hearing loss if better-ear PTA ≥35 dB HL and ≤90 dB HL. The total number of individuals reported in the table does not add to 3,602 (the total sample) because 7 Black participants and 67 White participants had missing data on some, but not all, hearing thresholds; these participants are included in the regression models after hearing status was imputed using multiple imputation. The P-trend value was obtained by modeling hearing loss categories as a continuous variable and is consistent with stronger associations between hearing loss and dementia as hearing loss severity increases. The P-interaction value is from the interaction term between race and hearing loss included in the model. Estimates in boldface are statistically significant at the alpha = 0.05 level.
